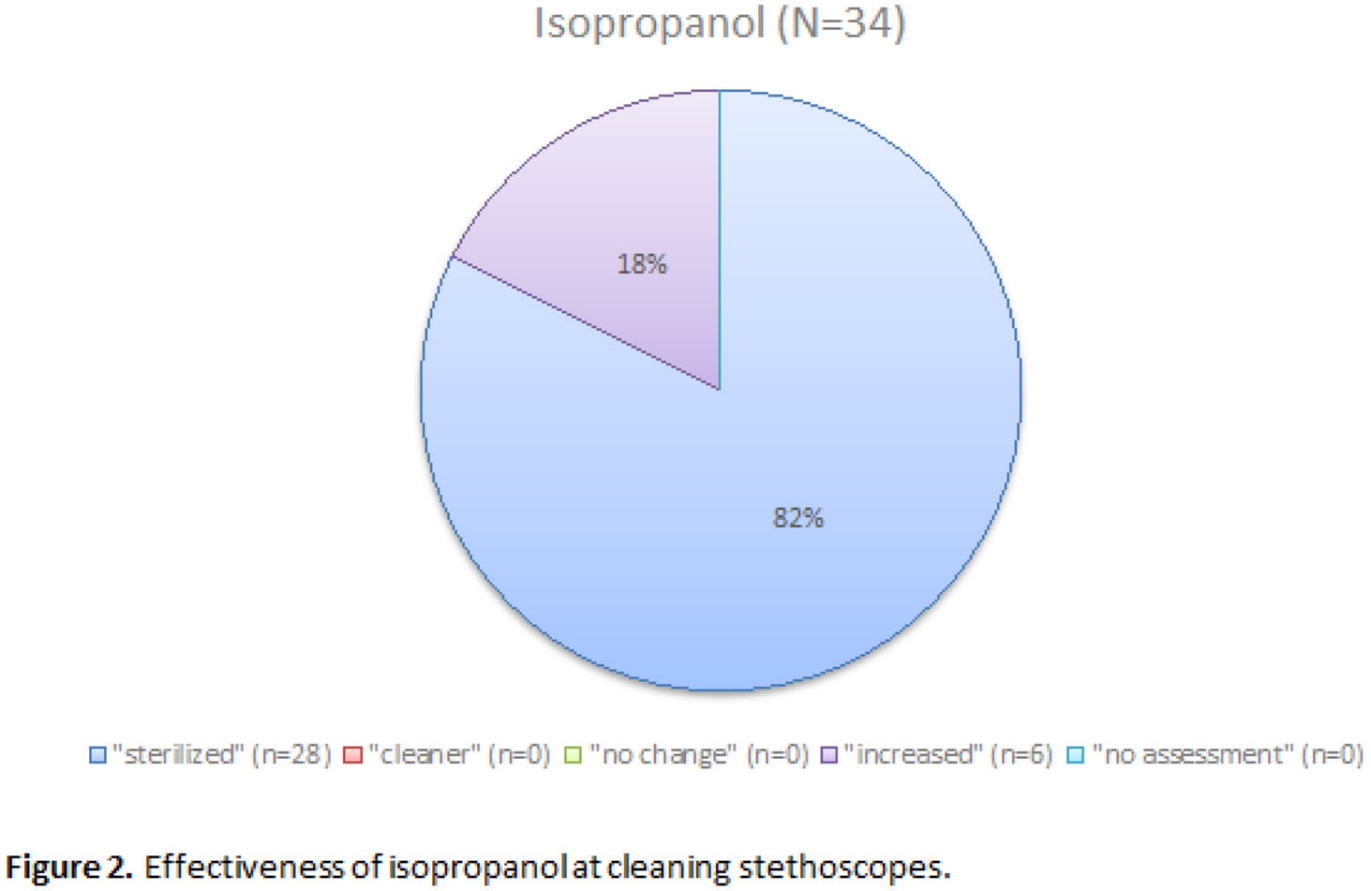# Assessment of cleaning stethoscopes using UV-C sanitation

**DOI:** 10.1017/ash.2022.143

**Published:** 2022-05-16

**Authors:** Austin Carmack, Niteesh Sundaram, Burt Cagir, Cathy Lanning, Kayla Robinson, Anne Rizzo

## Abstract

**Background:** It is well established that stethoscopes harbor pathogenic bacteria species. Within hospital settings, these pathogens can be rapidly transmitted from room to room and can cause harm in vulnerable populations. The current literature demonstrates that disinfecting stethoscopes with isopropanol kills 99% of all bacteria. However, in practice this rarely occurs and disinfection is subject to user error. We assessed the efficacy of ultraviolet germicidal irradiation (UV-C) at decontaminating stethoscopes used at our rural healthcare system along with the cleaning habits of their users. **Methods:** Stethoscopes were randomly selected from the clinical staff of our hospital’s largest nursing unit. The stethoscopes were each swabbed for culture then exposed to UV-C for 20 seconds and sampled again. Users were asked to complete a survey during this process. Samples were then cultivated on tryptone soya broth (TSB) agar, and all growth was sent for identification via matrix-assisted laser desorption/ionization (MALDI-TOF). Later, the protocol was repeated to assess cleaning efficacy of the isopropanol wipes commonly used in our hospital. We collected pre- and postintervention samples after cleaning vigorously for 3 minutes according to the manufacturer’s guidelines. The samples were classified as follows: “cleaner” if the number of colonies decreased after sanitation, “sterilized” if the number of colonies decreased to zero, “no change” if the number of colonies stayed the same, and “no assessment” if there was no preintervention growth. Several samples “increased” in CFU count after the intervention, likely due to incomplete sampling, contamination, or incomplete penetration of UV-C. The Fisher exact test was used to analyze the effectiveness of the stethoscope sanitation techniques. **Results:** In total, 60 samples (33 used for analysis) were obtained from stethoscopes cleaned with UV-C (Fig. 1). Moreover, 34 samples (28 used for analysis) were obtained from stethoscopes cleaned with isopropanol (Fig. 2). Both UV-C (93.9% vs 6.1%; *P* < .01) and isopropanol (100% vs 0%; *P* < .01) resulted in a significant decrease in bacterial colonization on stethoscopes. UV-C was not more effective at sanitizing stethoscopes than isopropanol (93.9% vs 100%; *P* = .50). **Conclusions:** Both UV-C and isopropanol were effective at cleaning hospital stethoscopes. Given that UV-C is not subject to user error and that it takes less time to clean a stethoscope than isopropanol, it may be the superior option in a clinical setting.

**Funding:** None

**Disclosures:** None

**Figure:**
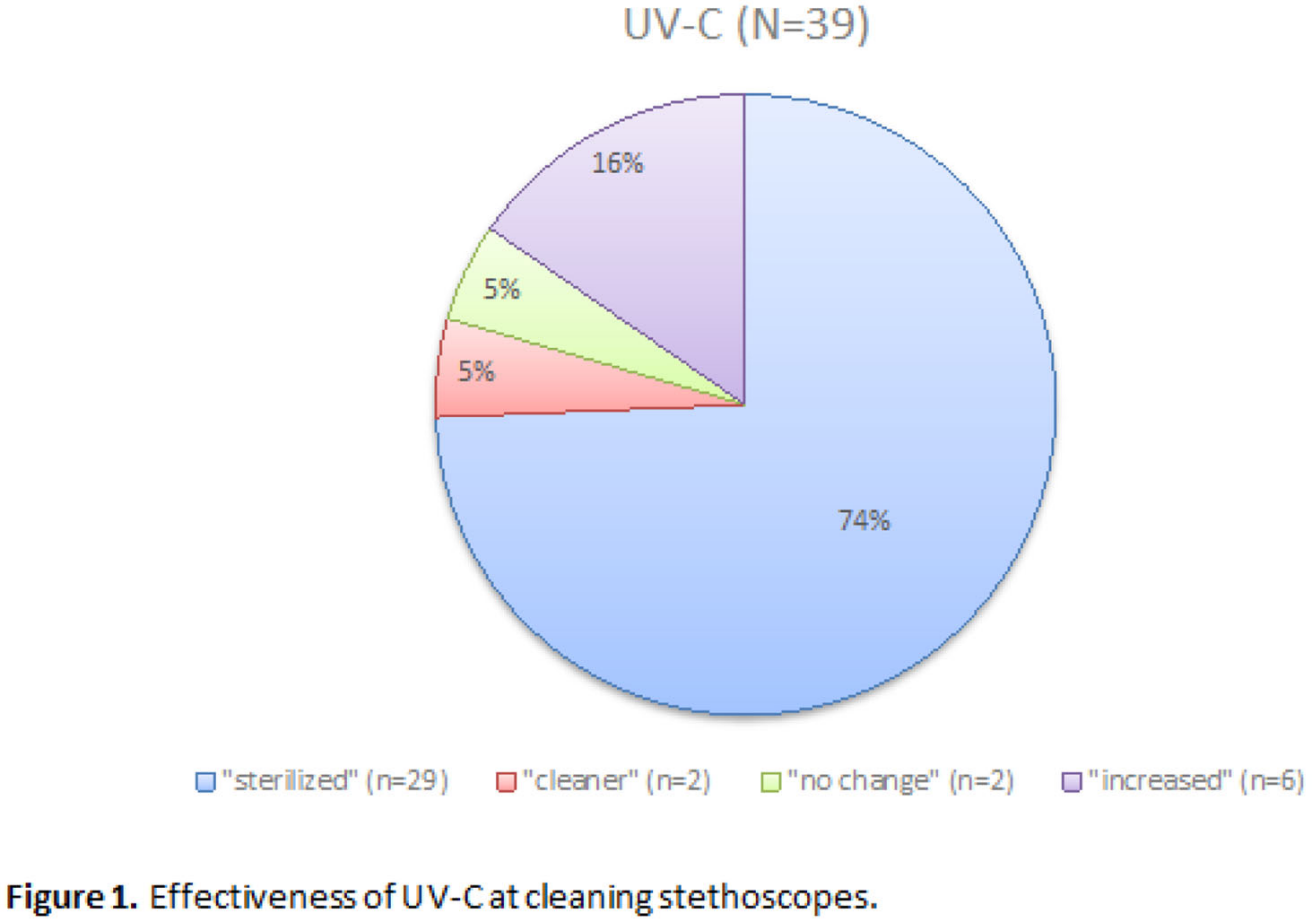

**Figure:**